# A systematic classification of death causes in multiple myeloma

**DOI:** 10.1038/s41408-018-0068-5

**Published:** 2018-03-08

**Authors:** Elias K. Mai, Eva-Maria Haas, Stephan Lücke, Martin Löpprich, Christina Kunz, Maria Pritsch, Petra Knaup-Gregori, Marc S. Raab, Jana Schlenzka, Uta Bertsch, Jens Hillengass, Hartmut Goldschmidt

**Affiliations:** 1 Department of Internal Medicine V, University Hospital Heidelberg, Heidelberg, Germany; 2 National Center for Tumor Diseases (NCT) Heidelberg, Trail Center, Heidelberg, Germany; 3 Institute of Medical Biometry and Informatics, University of Heidelberg, Heidelberg, Germany; 4 Division of Biostatistics, German Cancer Research Center (DKFZ), Heidelberg, Germany; 5 National Center for Tumor Diseases (NCT) Heidelberg, Heidelberg, Germany

The introduction of high-dose therapy followed by autologous blood stem cell transplantation (HDT/ABSCT) and the novel agents led to continuously improving survival in multiple myeloma (MM)^1,2^. However, the majority of MM patients ultimately relapses or progresses, and deceases of disease related conditions such as severe infections, renal failure or toxicity^3,4^. Nonetheless, the increasing life expectancy of the MM population might translate into an increase in causes of death (COD) unrelated to MM.

The aim of this study was to develop and apply a reliable systematic classification for COD in MM, combining the assessment of a specific COD and a causal link between the COD and MM, MM therapy or unrelated conditions, and to assess the impact of known MM prognostic factors on COD.

A number of 818 MM patients, who had received an upfront HDT/ABSCT between June 1992 and October 2013 at the University Hospital Heidelberg, Heidelberg, Germany were included in this single-center, retrospective analysis (Supplemental Table 1). Until April 2014, 483 of the eligible patients were deceased. The median overall survival of the cohort was 5.9 years (Supplemental Figure 1). This analysis was approved by the ethics committee of the University of Heidelberg (Number S-337/2009). All patients gave written informed consent.

To construct a systematic COD classification, the process of qualitative content analysis was applied using MAXQDA (VERBI GmbH, Berlin, Germany) (Supplemental Figure 2)^5,6^. A preliminary classification was developed based on the results of a PubMed literature search on COD in MM and existing COD classifications^7,8^. Next, the preliminary COD classification (Supplemental Figure 3) was examined on the collective to evaluate feasibility and practicality. Based on this initial evaluation, a final, systematic COD classification for MM (Fig. 1) and a corresponding rule-based allocation algorithm (Supplemental Figure 4) were built and applied to our cohort.

**Fig. 1:**
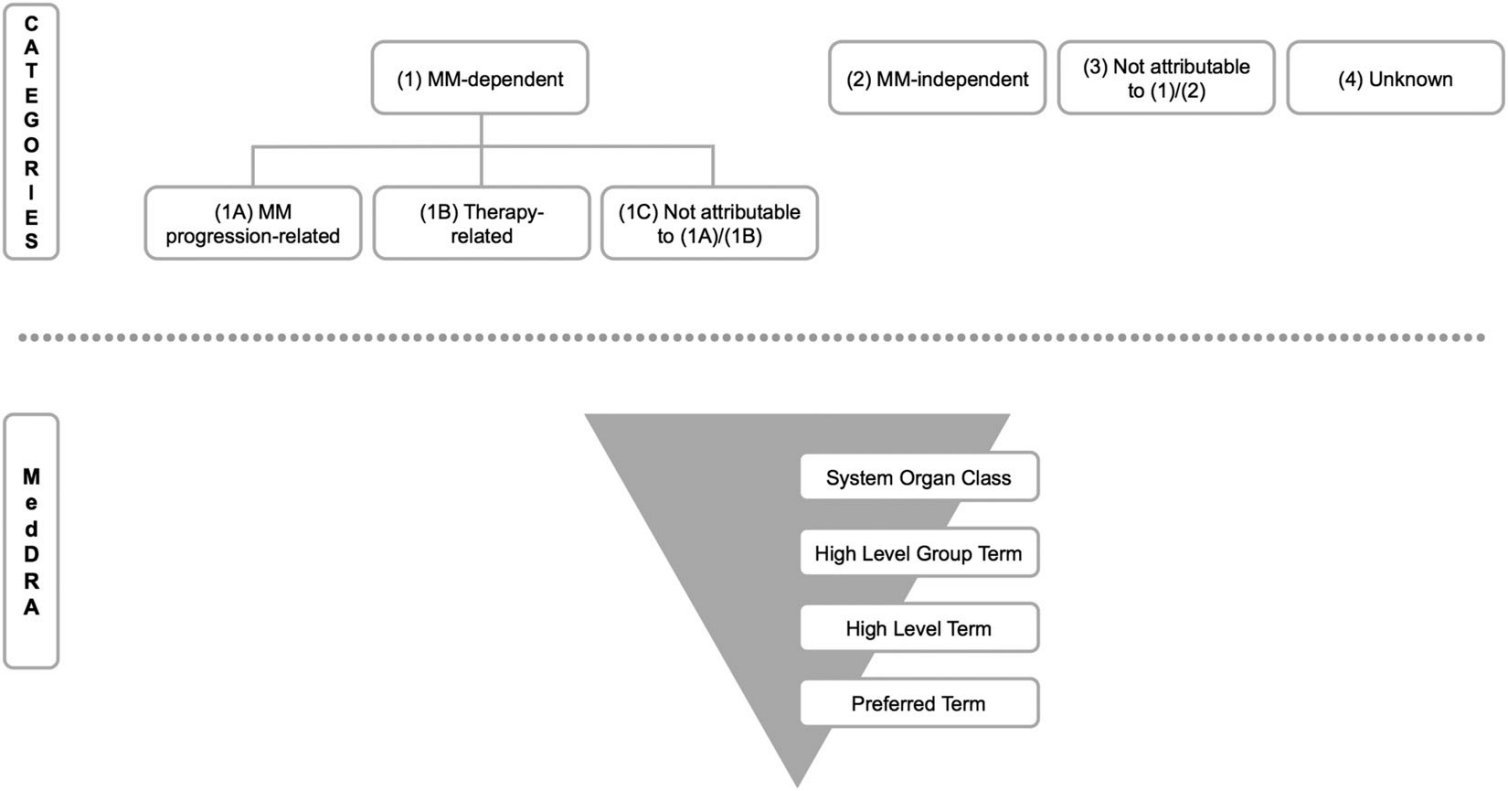
Proposed COD classification for MM. The classification consists of an hierarchical structure. The superordinate system allows the assignment of COD to MM, MM therapy or unrelated conditions. The subordinate system allows the specific assignment of an COD according to the MedDRA terminology

The final COD classification is hierarchically and systematically structured (Fig. 1). A superordinate system of categories determines the causal link between the COD and MM or side effects of MM therapy and distinguishes the following categories: (1) MM-dependent, (2) MM-independent, (3) not attributable to *(1)/(2)*, and (4) unknown (Fig. 1). In addition, MM-dependent COD (1) are subdivided into (1A) MM progression-related, (1B) therapy-related, and (1C) not attributable to *(1A)/(1B)*.

The subordinate system defines COD at four levels of different specificity applying the MedDRA terminology:^9^ the System Organ Class (SOC) has the lowest and the Preferred Term (PT) the highest specificity (Fig. 1).

According to the developed algorithm, COD were allocated to the different categories by consulting the available medical documentation within 90 days before death (Supplemental Figure 4). Examples for each category can be found in the Supplemental Methods.

Additionally, a validity system was developed and applied to the cohort to evaluate the reliability of the consulted medical documentation (Supplemental Methods). Statistical analyses were conducted using R version 3.2.218 and survival package (Supplemental Methods).

Among 483 deaths in our cohort, 80.7% (*n* = 390) were MM-dependent (1), 1.7% (*n* = 8) MM-independent (2), 7.0% (*n* = 34) not attributable to *(1)/(2)* (3), and 10.6% (*n* = 51) unknown (4) (Fig. 2a). The most common MM-independent (2) COD (*n* = 8) depicted in the lowest specific MedDRA term SOC were cardiac disorders (50.0%, *n* = 4) and nervous system disorders (25.0%, *n* = 2). In category (3) (*n* = 34) infections and neoplasms (both 26.5%, *n* = 9), cardiac disorders (20.6%, *n* = 7), nervous system disorders (8.8%, *n* = 3), and respiratory disorders (5.9%, *n* = 2) were the most frequent SOCs.

**Fig. 2:**
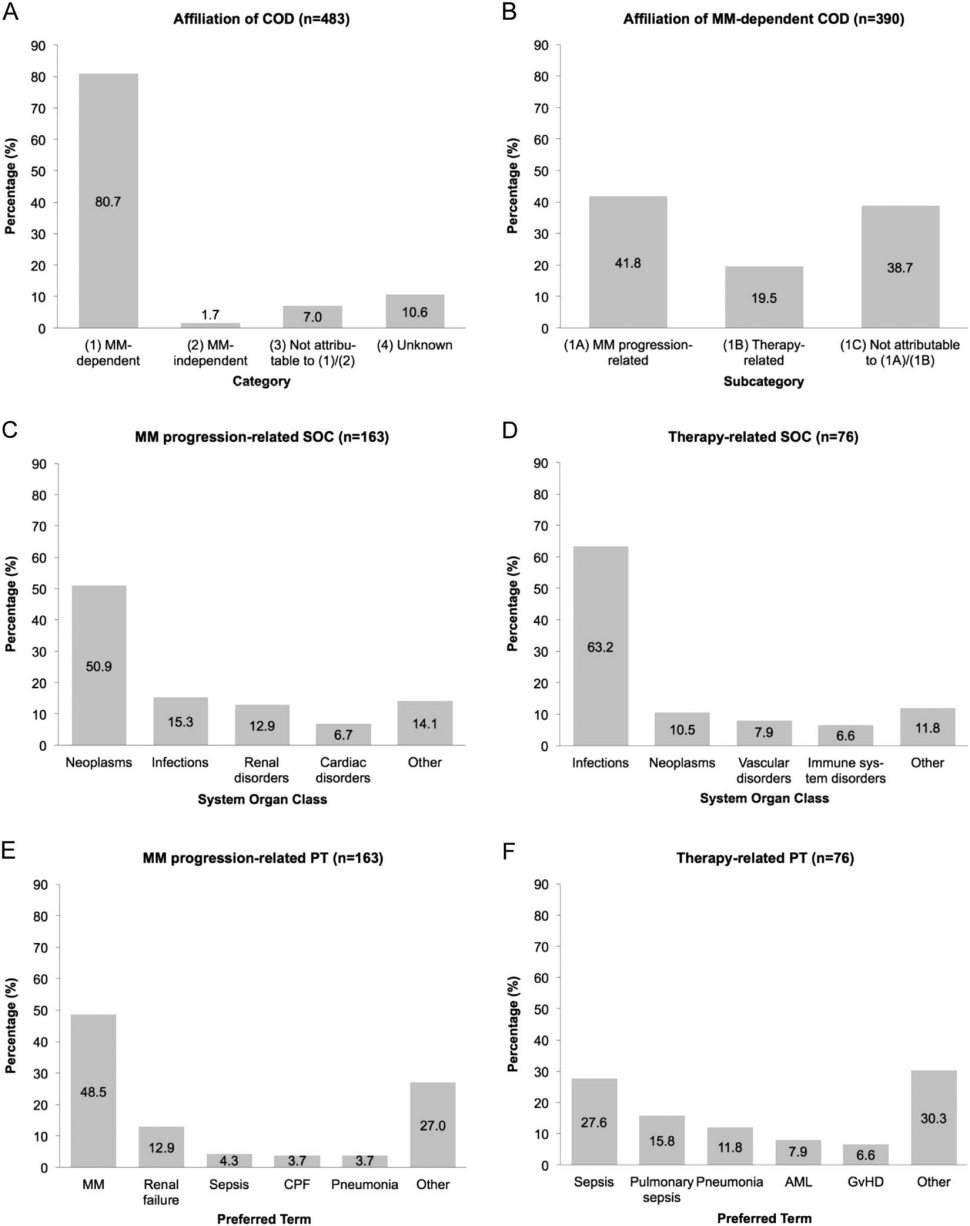
Frequency distribution of assigned death causes at different classification levels. **a** Categories. **b** Subcategories. **c** MM progression-related SOC. **d** Therapy-related SOC. **e** MM-progression-related PT. **f** Therapy-related PT

Among 390 MM-dependent (1) COD, 41.8% (*n* = 163) were MM progression-related (1A), 19.5% (*n* = 76) therapy-related (1B), and 38.7% (*n* = 151) not attributable to *(1A)/(1B)* (1C) (Fig. 2b). The most common MM progression-related (1A) SOCs (*n* = 163) were neoplasms (50.9%, *n* = 83), infections (15.3%, *n* = 25), renal disorders (12.9%, *n* = 21), and cardiac disorders (6.7%, *n* = 11, Fig. 2c). Expressed in the high specific MedDRA term PT, MM (48.5%, *n* = 79), renal failure (12.9%, *n* = 21), sepsis (4.3%, *n* = 7) and pneumonia as well as cardiopulmonal failure (3.7%, *n* = 6 each) were leading (Fig. 2e). Within the therapy-related (1B) category (*n* = 76), the most common SOC was infection (63.2%, *n* = 48) followed by neoplasms (10.5%, *n* = 8), vascular disorders (7.9%, *n* = 6), and immune system disorders (6.6%, *n* = 5) (Fig. 2d). The most common PTs were sepsis (27.6%, *n* = 21), pulmonary sepsis (15.8%, *n* = 12), pneumonia (11.8%, *n* = 9), acute myeloid leukemia (AML) (7.9%, *n* = 6), and Graft-versus-host-disease (GvHD) (6.6%, *n* = 5) (Fig. 2f).

In total, 17 of 483 (3.5%) deaths were caused by second primary malignancies (SPM). Eight hematologic SPM were assigned to the MM-dependent subcategory therapy-related (1B) and nine solid SPM to category (3) (Supplemental Table 2).

The temporal distribution of MM-dependent COD between 1994 and 2014 as well as after the first HDT/ABSCT is shown in Supplemental Figure 5. The results of the applied validity system are presented in Supplemental Figure 6.

In multivariate competing-risks analyses, age ≥ 65 years (HR = 1.85, *p* = 0.02), International Staging System (ISS) III (HR = 1.98, *p* = 0.01) and platelet counts <150/nl (HR = 2.37, *p* = 0.01) were linked to an increased risk for MM progression-related (1 A) death, with a trend for lactate dehydrogenase (LDH) ≥ 248 U/l (HR = 1.69, *p* = 0.06) being influential, too. Likewise, a low platelet count was associated with an increased risk for therapy-related (1B) death (HR = 2.87, *p* = 0.01). Renal impairment (RI, serum creatinine ≥ 2 mg/dl) showed a trend of negatively impacting the risk for therapy-related (1B) death (HR = 2.13, *p* = 0.11), though the effect was not significant (Supplemental Table 3).

Our current study is the first to describe a validated, rule-based and systematic classification for COD in MM. The definition of COD on different levels of specificity and the evaluation of the causal link between COD and MM or side effects of therapy are the core elements of the constructed classification, revealing parallels to existing COD classifications. A hierarchical structure can be found in the ICD-10 classification, allowing both an overview and a detailed determination of COD^7^. The classification of treatment-related mortality in children with cancer focuses on differentiating between treatment-related and -unrelated mortality^8^. Additionally, our classification allows a further discrimination of COD unrelated to treatment and contains categories for COD ambiguously associated with MM or side effects of therapy.

Before applying the presented classification in prospective MM trials or registries, a prospective validation is desirable. Therefore, the classification is currently applied in the prospective multicenter phase III trial of the German-speaking Myeloma Multicenter Group (GMMG)-HD6 [EUDRA-CT No. 2014-003079-40].

Among the known COD in our cohort, the vast majority was MM disease-related (90.2%, *n* = 390), whereas only a few cases were definitively unrelated to MM (1.9%, *n* = 8). This is in line with other studies^10,11^ and prompts that despite the improved prognosis in MM, the disease remains largely incurable.

Besides MM progression, infections (15.3%), renal disorders (12.9%), and cardiac disorders (6.7%) were the most common MM progression-related COD. In an analysis conducted by Riccardi and colleagues, infections and renal insufficiency constituted the leading MM-related COD, too^12^.

Infections (63.2%), SPM (10.5%), vascular disorders (7.9%), and GvHD (6.6%) were the leading therapy-related COD in our cohort. Bringhen and colleagues observed similar therapy-related COD, with infections, cardiac complications, SPM and venous thromboembolism being the most common^3^.

These findings prompt the early clinical evaluation and anti-infective treatment in MM patients undergoing MM therapy. However, studies on antibiotic or antiviral prophylaxis during MM therapy are rare. Additional preventive strategies for infectious complications should be further evaluated, such as the administration of polyvalent intravenous immunoglobulins.

SPM accounted for 3.5% of the death cases in the present study, which is in line with the results from a large meta-analysis (3.8%)^10^. Since MM, therapy, behavior, environment, and host-related factors play a role in the development of SPM, lethal solid SPM were assigned to category (3) in our study^4^. Due to melphalan exposure, lethal AML and myelodysplastic syndrome (MDS) were classified as therapy-related COD based on the AML classification of the World Health Organization^13^. However, Mailankody and colleagues were able to demonstrate that patients with monoclonal gammopathy of undetermined significance (MGUS) have an eight-fold elevated risk for the development of AML/MDS, too, although they did not receive MM therapy^14^.

Our competing risks analyses indicate for the first time that RI and thrombocytopenia constitute important risk factors for therapy-related death. Possibly because these patients seem to be at risk for severe side effects of MM therapy and perhaps have an impaired ability to recover. Initial RI might also hint at existing comorbidities, such as diabetes mellitus. Initial thrombocytopenia demonstrates restricted hematopoiesis due to high tumor burden^15^. An additional therapy-induced myelosuppression might lead to a substantially increased risk for corresponding complications, such as infections or bleedings. However, our analysis indicated that RI and thrombocytopenia also remain significant risk factors for MM progression-related death as described earlier^15^.

To conclude, the majority of transplant-eligible MM patients died MM-dependent, with one-fifth of these cases caused by therapeutic side effects. Infections, SPM and GvHD were the most common therapy-related COD. Among MM progression-related COD infections, renal failure and cardiopulmonary failure were leading. Competing risks analyses revealed RI and thrombocytopenia as important risk factors for therapy-related death. Finally, our constructed classification has proved to be reliable in the present analysis. Its future implementation in registries or prospective trials, such as the randomized phase III trial GMMG-HD6, might enable generating reliable data on COD in MM patients.

## Electronic supplementary material

Supplemental Table 1

Supplemental Table 2

Supplemental Table 3

Supplemental Material

Supplemental Figure 1

Supplemental Figure 2

Supplemental Figure 3

Supplemental Figure 4

Supplemental Figure 5

Supplemental Figure 6
