# Supplementary material for: A systematic classification of death causes in multiple myeloma
Source: Blood Cancer J. 2018 Mar 8;8(3):30. doi: 10.1038/s41408-018-0068-5 (PMC5843652; doi:10.1038/s41408-018-0068-5)
Supplement: Supplementary file 1 — Supplemental Table 1 [file 41408_2018_68_MOESM1_ESM.docx]

| **Supplemental Table 1.**  Baseline characteristics at diagnosis. | | | |
| --- | --- | --- | --- |
| Characteristic | n | | % |
| Sex |  | |  |
| Male | 491 | | 60.0 |
| Female | 327 | | 40.0 |
| Age (years) |  | |  |
| < 65 | 690 | | 84.3 |
| ≥ 65 | 128 | | 15.6 |
| Heavy chain isotype | | |  |
| IgG | | 434 | 53.1 |
| IgA | | 173 | 21.1 |
| Bence Jones | | 151 | 18.5 |
| Other | | 55 | 6.7 |
| Missing | | 5 | 0.6 |
| Light chain isotype | | |  |
| Kappa | | 510 | 62.4 |
| Lambda | | 284 | 34.7 |
| Kappa/Lambda | | 1 | 0.1 |
| Missing | | 23 | 2.8 |
| ISS | |  |  |
| I | | 266 | 32.5 |
| II | | 194 | 23.7 |
| III | | 152 | 18.6 |
| Missing | | 206 | 25.2 |
| LDH (U/l) | |  |  |
| < 248 | | 487 | 59.5 |
| ≥ 248 | | 94 | 11.5 |
| Missing | | 237 | 29.0 |
| Calcium (mmol/l) | | |  |
| ≤ 2.65 | | 516 | 63.1 |
| > 2.65 | | 95 | 11.6 |
| Missing | | 207 | 25.3 |
| Creatinine (mg/dl) | | |  |
| ≤ 2 | | 565 | 69.1 |
| > 2 | | 98 | 12.0 |
| Missing | | 155 | 18.9 |
| Hemoglobin (g/dl) | | |  |
| ≤ 10 | | 198 | 24.2 |
| > 10 | | 440 | 53.8 |
| Missing | | 180 | 22.0 |
| Platelets (per nl) | | |  |
| < 150 | | 56 | 6.8 |
| ≥ 150 | | 468 | 57.2 |
| Missing | | 294 | 35.9 |
| Novel agents in IT | |  |  |
| Thalidomide | | 98 | 12.0 |
| Bortezomib | | 201 | 24.6 |
| Lenalidomide | | 15 | 1.8 |
| No novel agent | | 523 | 63.9 |
| Abbreviations: ISS, International Staging System; IT, Induction Therapy; LDH, lactate dehydrogenase. | | | |
