# Supplementary material for: A systematic classification of death causes in multiple myeloma
Source: Blood Cancer J. 2018 Mar 8;8(3):30. doi: 10.1038/s41408-018-0068-5 (PMC5843652; doi:10.1038/s41408-018-0068-5)
Supplement: Supplementary file 2 — Supplemental Table 2 [file 41408_2018_68_MOESM2_ESM.docx]

| **Supplemental Table 2.** Fatal second primary malignancies. | | | |
| --- | --- | --- | --- |
| **(1B) Therapy-related** | | **(3) Not attributable to (1)/(2)** | |
| **Hematologic SPM** | | **Solid SPM** | |
| PT | n | PT | n |
| Acute myeloid leukemia | 6 | Glioblastoma | 2 |
| Myelodysplastic syndrome | 1 | Adenocarcinoma lung | 1 |
| Non-Hodgkin lymphoma | 1 | Cholangiocarcinoma | 1 |
|  |  | Metastatic breast cancer | 1 |
|  |  | Metastatic colon cancer | 1 |
|  |  | Metastatic melanoma | 1 |
|  |  | Metastatic stomach cancer | 1 |
|  |  | NSCLC | 1 |
| Total hematologic | 8 | Total solid | 9 |
| 17 fatal SPM in our cohort, representing 3.5% of all cases of death. Abbreviations: NSCLC, Non-Small Cell Lung Cancer; PT, Preferred Term; SPM, second primary malignancy. | | | |
