# Supplementary material for: A systematic classification of death causes in multiple myeloma
Source: Blood Cancer J. 2018 Mar 8;8(3):30. doi: 10.1038/s41408-018-0068-5 (PMC5843652; doi:10.1038/s41408-018-0068-5)
Supplement: Supplementary file 3 — Supplemental Table 3 [file 41408_2018_68_MOESM3_ESM.docx]

| **Supplemental Table 3.** Results of the competing-risks analyses: impact of parameters on the hazard of dying MM-progression related versus therapy-related. | | | | |
| --- | --- | --- | --- | --- |
|  | Univariate competing-risks analysis | | | |
|  | MM-progression related | | Therapy-related | |
| Parameter | HR (95%-CI) | P value | HR (95%-CI) | P value |
| Age (≥ 65 years) | 1.28 (0.83 – 1.97) | 0.27 | 1.25 (0.67 – 2.33) | 0.49 |
| ISS (III) | 2.26 (1.57 – 3.24) | **<0.001** | 1.41 (0.80 – 2.46) | 0.23 |
| LDH (≥ 248 U/l) | 2.16 (1.39 – 3.34) | **0.001** | 1.23 (0.60 – 2.50) | 0.57 |
| Creatinine (≥ 2 mg/dl) | 2.13 (1.41 – 3.22) | **<0.001** | 1.95 (1.04 – 3.64) | **0.04** |
| Platelet count (< 150/nl) | 2.61 (1.53 – 4.44) | **<0.001** | 2.53 (1.19 – 5.39) | **0.02** |
|  | Multivariate competing-risks analysis | | | |
|  | MM-progression related | | Therapy-related | |
| Parameter | HR (95%-CI) | P value | HR (95%-CI) | P value |
| Age (≥ 65 years) | 1.85 (1.09 – 3.13) | **0.02** | 1.17 (0.52 – 2.65) | 0.71 |
| ISS (III) | 1.98 (1.18 – 3.31) | **0.01** | 0.71 (0.30 – 1.68) | 0.44 |
| LDH (≥ 248 U/l) | 1.69 (0.97 – 2.95) | 0.06 | 1.46 (0.65 – 3.30) | 0.36 |
| Creatinine (≥ 2 mg/dl) | 1.10 (0.57 – 2.14) | 0.77 | 2.13 (0.85 – 5.34) | 0.11 |
| Platelet count (< 150/nl) | 2.37 (1.24 – 4.53) | **0.01** | 2.87 (1.26 – 6.51) | **0.01** |
| Abbreviations: CI, confidence interval; HR, hazard ratio; ISS, International Staging System; LDH, lactate dehydrogenase; MM, multiple myeloma. | | | | |
