# Supplementary material for: A systematic classification of death causes in multiple myeloma
Source: Blood Cancer J. 2018 Mar 8;8(3):30. doi: 10.1038/s41408-018-0068-5 (PMC5843652; doi:10.1038/s41408-018-0068-5)
Supplement: Supplementary file 4 — Supplemental Material [file 41408_2018_68_MOESM4_ESM.docx]

**Supplemental material**

A systematic classification of death causes in multiple myeloma

Elias K. Mai^1^*, Eva-Maria Haas^1^*, Stephan Lücke^2^, Martin Löpprich^3^, Christina Kunz^4^, Maria Pritsch^1^, Petra Knaup-Gregori^3^, Marc S. Raab^1^, Jana Schlenzka^1^, Uta Bertsch^1^, Jens Hillengass^1^ and Hartmut Goldschmidt^1,5^

^1^Department of Internal Medicine V, University Hospital Heidelberg, Heidelberg, Germany; ^2^National Center for Tumor Diseases (NCT) Heidelberg, Trail Center, Heidelberg, Germany; ^3^Institute of Medical Biometry and Informatics, University of Heidelberg, Heidelberg, Germany; ^4^Division of Biostatistics, German Cancer Research Center (DKFZ), Heidelberg, Germany and ^5^National Center for Tumor Diseases (NCT) Heidelberg, Heidelberg, Germany.

** These authors contributed equally to this work.*

**Supplemental Methods**

Examples for the allocation of COD to categories

*Example 1:* The follow-up revealed that patient 1 has died at home in 2016. He has not performed his regular MM check ups at the University Hospital Heidelberg for the last two years. His general practitioner did not have information about the COD and an autopsy was not conducted.

**-> Unknown (4)**

*Example 2:* Patient 2 was brought to the hospital by his daughter, after he had been fallen. The laboratory examinations showed a thrombocytopenia and a progression of MM. A CT scan of the head showed a subdural hematoma, which finally led to death.

**-> Not attributable to *(1)/(2)* (3)**

*Example 3:* Patient 3 had a known alcohol abuse with subsequent liver cirrhosis. He developed an upper gastrointestinal bleeding, which led to death. At the time of death MM was in complete response and no MM therapy was applied.

**-> MM-independent (2)**

*Example 4:* Patient 4 was hospitalized because of a reduced general condition and somnolence. In the past, several MM therapies had been conducted. The laboratory examinations showed increased creatinine values, anemia, thrombocytopenia and a rapid MM progression. Due to lack of therapeutic options a palliative strategy was initiated in agreement with the patient and his relatives. In the next days the creatinine values increased further. Finally, the patient died due to renal failure.

**-> MM-dependent (1) -> MM progression-related (1A)**

*Example 5:* After the MM control examination had shown progressive disease, a reinduction therapy with Bortezomib-Cyclophosphamide-Dexamethasone was initiated in this patient. However, two weeks later patient 5 developed cough, dyspnea and fever. The laboratory examinations showed increased inflammation parameters. Despite of antibiotic therapy he became respiratory insufficient and died due to pneumonia.

**-> MM-dependent (1), not attributable to *(1A)/(1B)***

*Example 6:* Patient 6 developed dyspnea and fever two weeks after HDT/ABSCT. Prior tho HDT/ABSCT his MM disease had well responded to upfront indcution therapy. A conducted chest X-ray showed a basal infiltrate on the right side. An antibiotic therapy with ceftazidim was initiated and escalated with an antimykotic therapy. Nevertheless, the patient became respiratory insufficient, needed catecholamines and died due to pulmonary sepsis.

**-> MM-dependent (1) -> therapy-associated (1B)**

*Example 7:* Patient 7 developed GvHD of the intestine and the liver after allogeneic blood stem cell transplantation after MM relapse and re-induction therapy. The symptoms were progressive despite of high dose immunosuppressive therapy. In agreement with the patient no intensive care interventions were conducted. The patient died due to GvHD.

**-> MM-dependent (1) -> therapy-associated (1B)**

Validity system

The sources of medical documentation were rated in two categories: death certificates and documents containing information confirmed by physicians received a high validity whereas documents or information created by non-physicians received a low validity.

Statistical analyses

The COD frequencies were analyzed at different super- (causality to MM) and subordinate (MedDRA specific COD) levels of the classification. Uni- and multivariate competing-risks analyses were performed on the whole cohort (n=818) to assess the impact of the known MM prognostic factors (dichotomized) on the combined endpoint COD/overall survival: age (≥65 years), International Staging System (ISS, stage III), lactate dehydrogenase (LDH, ≥248 U/ml) and platelet count at diagnosis (<150/nl) as well as renal impairment (RI) at diagnosis or prior to ABSCT (serum creatinine ≥2mg/dl). Analyses were restricted to the subset of patients with unique COD either MM progression-related (1A) or therapy-related death (1B), plus all patients alive at the end of observation. The impact of covariates was assessed by fitting multi-state models. Using three-state models, patients enter the analysis in an event-free state (alive), and are then considered to be under risk of dying from either one of two competing causes (1A vs. 1B); both endpoints are mutually exclusive, only one can be observed. Upon their deaths, subjects switch into either one of the two final states. Cox regression on the cause-specific hazards was used to evaluate the specific impact of covariates on each of the two competing death risks. Hazard ratios (HR) were estimated with their respective 95% confidence interval; effects were considered statistically significant if p≤0.05.

**Supplemental Figure legends**

**Supplemental Figure 1.** Kaplan Meier estimate of the survival function with 95% CI. Median OS 5.87 years (5.16 - 6.49).

Abbreviations: ABSCT, autologous blood stem cell transplantation; CI, confidence interval; OS, overall survival.

**Supplemental Figure 2.** Process of developing a cause of death-classification according to the Qualitative Content Analysis.

**Supplemental Figure 3.** Hierarchical structure of the preliminary COD classification in MM patients.

Abbreviations: COD, causes of death; MM, multiple myeloma.

**Supplemental Figure 4.** Algorithm for allocation of COD within the superordinate system of the classification.

The algorithm allows an reproducable assignment of COD in relation to MM/therapy or unrelated conditions.

Abbreviations: ABSCT, autologous blood stem cell transplantation; COD, causes of death; GvHD, graft versus host disease; MM, multiple myeloma.

**Supplemental Figure 5.** Affiliation of MM-dependent death causes in periods. (A) Periods 1994-2000, 2001-2007 and 2008-2014. (B) Periods ≤6 months, >6-72 months and >72 months after the first ABSCT.

Abbreviations: ABSCT, autologous blood stem cell transplantation; COD, causes of death; MM, multiple myeloma.

**Supplemental Figure 6.** Frequency distribution of assigned death causes at different classification levels depending on validity. (A) Categories. (B) Subcategories. (C) MM-dependent SOC. (D) Not attributable SOC.

Abbreviations: COD, causes of death; MM, multiple myeloma; NS, nervous system; SOC, System Organ Class.
