# Supplementary material for: A systematic classification of death causes in multiple myeloma
Source: Blood Cancer J. 2018 Mar 8;8(3):30. doi: 10.1038/s41408-018-0068-5 (PMC5843652; doi:10.1038/s41408-018-0068-5)
Supplement: Supplementary file 5 — Supplemental Figure 1 [file 41408_2018_68_MOESM5_ESM.pdf]

Supplemental

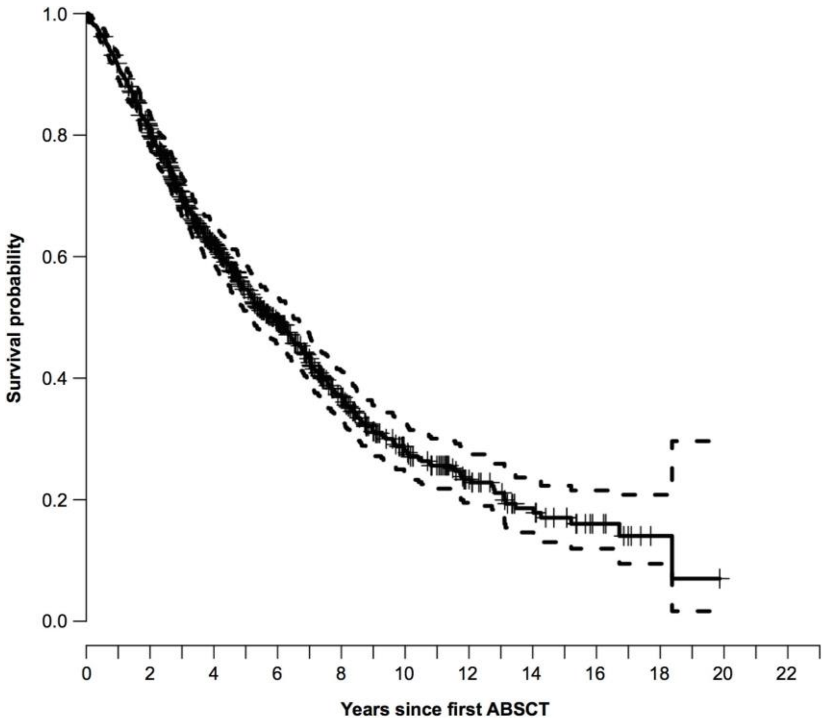

Fig S1. Kaplan Meier estimate of the survival function with 95% CI. Median OS 5.87 years (5.16 - 6.49). Abbreviations: ABSCT, autologous blood stem cell transplantation; CI, confidence interval; OS, overall survival.
