# Supplementary material for: A systematic classification of death causes in multiple myeloma
Source: Blood Cancer J. 2018 Mar 8;8(3):30. doi: 10.1038/s41408-018-0068-5 (PMC5843652; doi:10.1038/s41408-018-0068-5)
Supplement: Supplementary file 6 — Supplemental Figure 2 [file 41408_2018_68_MOESM6_ESM.pdf]

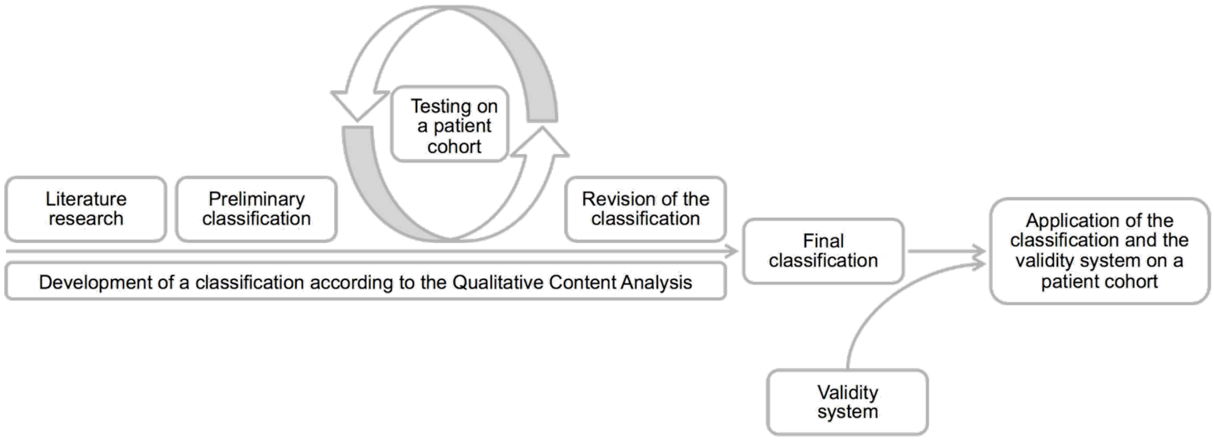

Fig S2. Process of developing a cause of death-classification according to the Qualitative Content Analysis.
