# Supplementary material for: A systematic classification of death causes in multiple myeloma
Source: Blood Cancer J. 2018 Mar 8;8(3):30. doi: 10.1038/s41408-018-0068-5 (PMC5843652; doi:10.1038/s41408-018-0068-5)
Supplement: Supplementary file 7 — Supplemental Figure 3 [file 41408_2018_68_MOESM7_ESM.pdf]

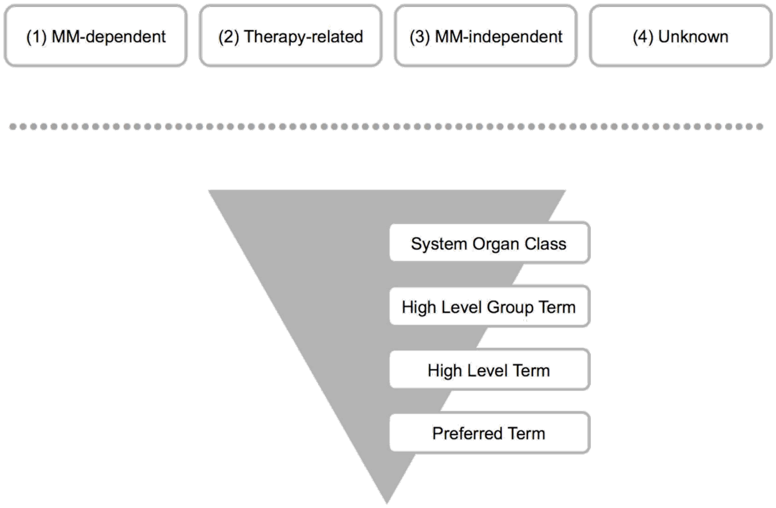

Fig S3. Hierarchical structure of the preliminary COD classification in MM patients. Abbreviations: COD, causes of death; MM, multiple myeloma.
