# Supplementary material for: A systematic classification of death causes in multiple myeloma
Source: Blood Cancer J. 2018 Mar 8;8(3):30. doi: 10.1038/s41408-018-0068-5 (PMC5843652; doi:10.1038/s41408-018-0068-5)
Supplement: Supplementary file 8 — Supplemental Figure 4 [file 41408_2018_68_MOESM8_ESM.pdf]

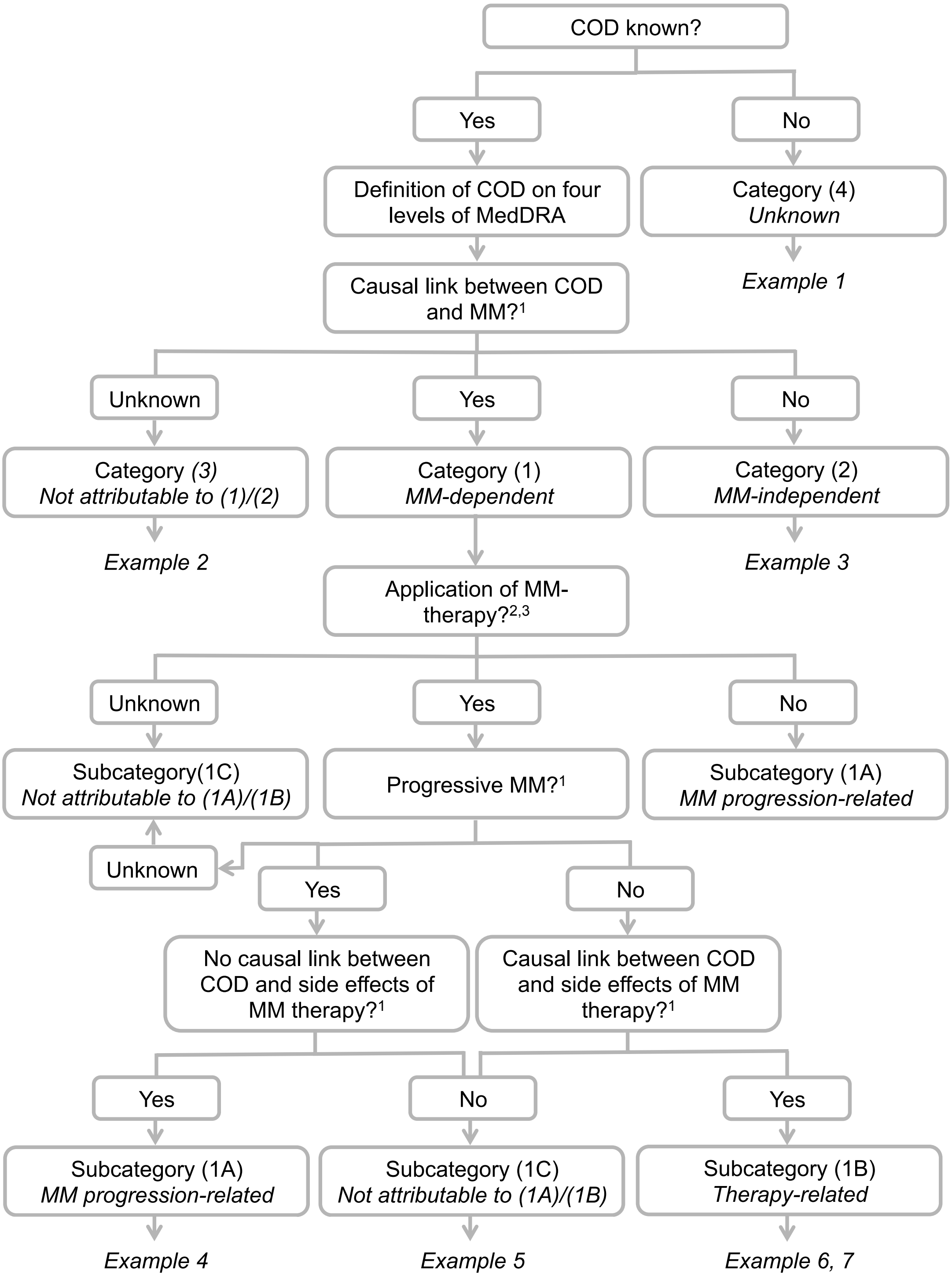

[1] According to available medical documentation within 90 days before death.

[2] MM-therapy: antitumor therapy, autologous and allogenic blood stem cell transplantation, radiotherapy and surgical measures. Palliative therapy excluded.

[3] Application of MM-therapy within 30 days before death according to available medical documentation.

Exceptions:
- Time limit 100 days after ABSCT
- No time limit for GvHD and therapy-related second primary malignancy
- Extended time limit for side effects with typical latency

Fig. S4. Algorithm for allocation of COD within the superordinate system of the classification. The algorithm allows an reproducable assignment of COD in relation to MM/therapy or unrelated conditions. Abbreviations: ABSCT, autologous blood stem cell transplantation; COD, causes of death; GvHD, graft versus host disease; MM, multiple myeloma.
