# Supplementary material for: A systematic classification of death causes in multiple myeloma
Source: Blood Cancer J. 2018 Mar 8;8(3):30. doi: 10.1038/s41408-018-0068-5 (PMC5843652; doi:10.1038/s41408-018-0068-5)
Supplement: Supplementary file 9 — Supplemental Figure 5 [file 41408_2018_68_MOESM9_ESM.pdf]

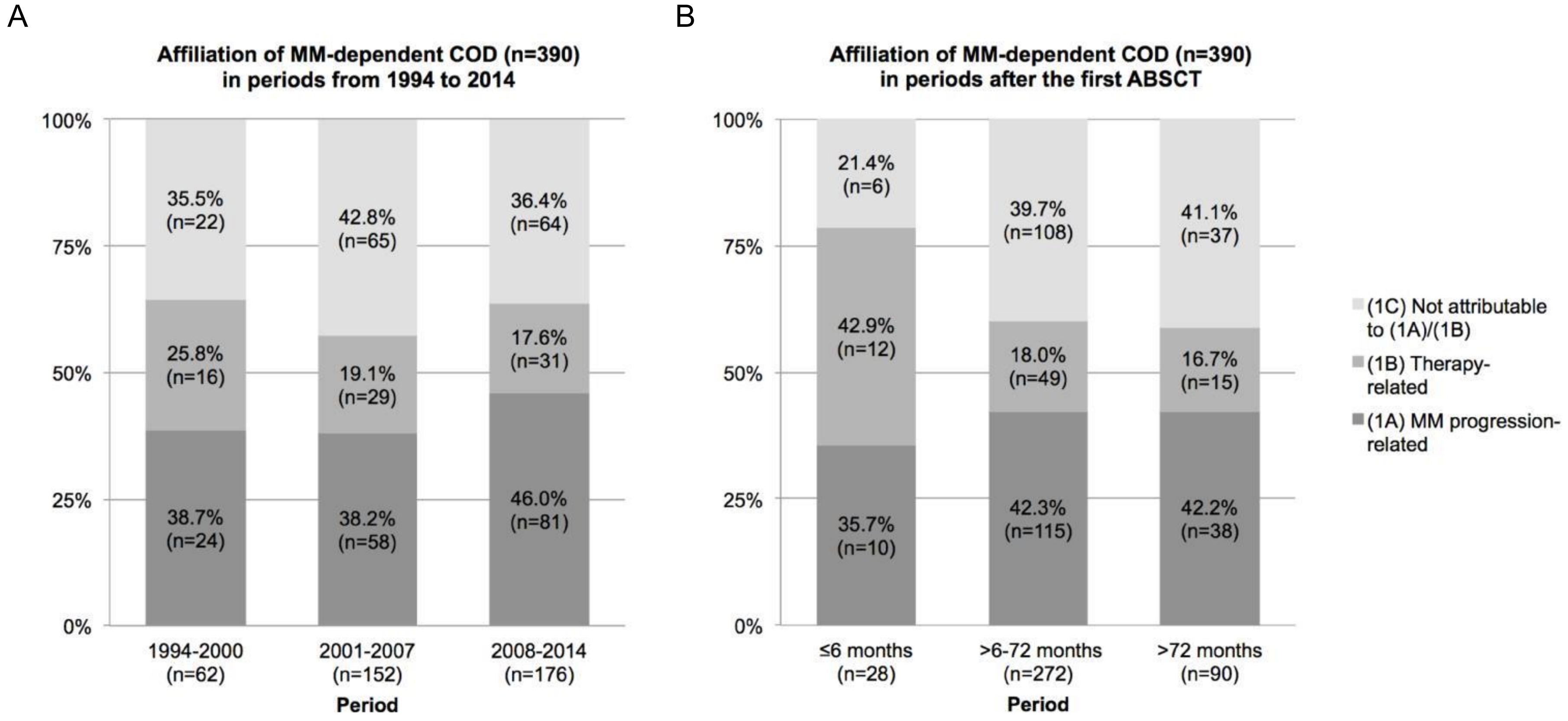

Fig. S5. Affiliation of MM-dependent death causes in periods. (A) Periods 1994-2000, 2001-2007 and 2008-2014. (B) Periods ≤6 months, >6-72 months and >72 months after the first ABSCT. Abbreviations: ABSCT, autologous blood stem cell transplantation; COD, causes of death; MM, multiple myeloma.
