# Supplementary material for: A systematic classification of death causes in multiple myeloma
Source: Blood Cancer J. 2018 Mar 8;8(3):30. doi: 10.1038/s41408-018-0068-5 (PMC5843652; doi:10.1038/s41408-018-0068-5)
Supplement: Supplementary file 10 — Supplemental Figure 6 [file 41408_2018_68_MOESM10_ESM.pdf]

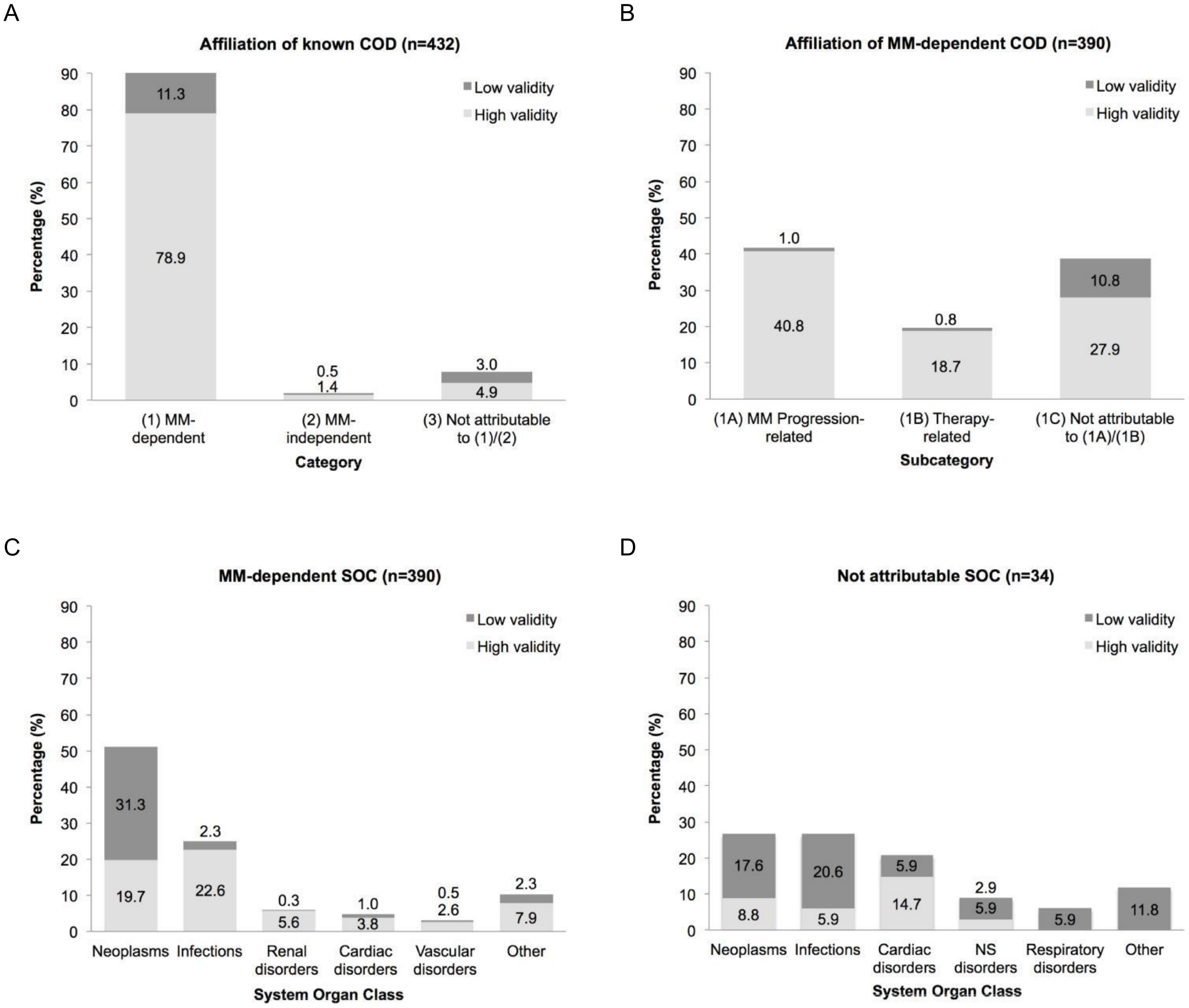

Fig S6. Frequency distribution of assigned death causes at different classification levels depending on validity. (A) Categories. (B) Subcategories. (C) MM-dependent SOC. (D) Not attributable SOC. Abbreviations: COD, causes of death; MM, multiple myeloma; NS, nervous system; SOC, System Organ Class.
